# Detection of *Legionella* species, the influence of precipitation on the amount of *Legionella* DNA, and bacterial microbiome in aerosols from outdoor sites near asphalt roads in Toyama Prefecture, Japan

**DOI:** 10.1186/s12866-021-02275-2

**Published:** 2021-07-17

**Authors:** Jun-ichi Kanatani, Masanori Watahiki, Keiko Kimata, Tomoko Kato, Kaoru Uchida, Fumiaki Kura, Junko Amemura-Maekawa, Junko Isobe

**Affiliations:** 1 Department of Bacteriology, Toyama Institute of Health, 17-1 Nakataikoyama, 939-0363 Imizu-city, Toyama, Japan; 2 Department of Bacteriology I, National Institute of Infectious Diseases, 1-23-1 Toyama, Shinjuku-ku, 162-8640 Tokyo, Japan

**Keywords:** *Legionella* species, Legionellosis, Aerosols, Asphalt roads, Microbiome

## Abstract

**Background:**

Legionellosis is caused by the inhalation of aerosolized water contaminated with *Legionella* bacteria. In this study, we investigated the prevalence of *Legionella* species in aerosols collected from outdoor sites near asphalt roads, bathrooms in public bath facilities, and other indoor sites, such as buildings and private homes, using amoebic co-culture, quantitative PCR, and 16S rRNA gene amplicon sequencing.

**Results:**

*Legionella* species were not detected by amoebic co-culture. However, *Legionella* DNA was detected in 114/151 (75.5%) air samples collected near roads (geometric mean ± standard deviation: 1.80 ± 0.52 log_10_ copies/m^3^), which was comparable to the numbers collected from bathrooms [15/21 (71.4%), 1.82 ± 0.50] but higher than those collected from other indoor sites [11/30 (36.7%), 0.88 ± 0.56] (*P* < 0.05). The amount of *Legionella* DNA was correlated with the monthly total precipitation (*r* = 0.56, *P* < 0.01). It was also directly and inversely correlated with the daily total precipitation for seven days (*r* = 0.21, *P* = 0.01) and one day (*r* = − 0.29, *P* < 0.01) before the sampling day, respectively. 16S rRNA gene amplicon sequencing revealed that *Legionella* species were detected in 9/30 samples collected near roads (mean proportion of reads, 0.11%). At the species level, *L. pneumophila* was detected in 2/30 samples collected near roads (the proportion of reads, 0.09 and 0.11% of the total reads number in each positive sample). The three most abundant bacterial genera in the samples collected near roads were *Sphingomonas*, *Streptococcus*, and *Methylobacterium* (mean proportion of reads; 21.1%, 14.6%, and 1.6%, respectively). In addition, the bacterial diversity in outdoor environment was comparable to that in indoor environment which contains aerosol-generating features and higher than that in indoor environment without the features.

**Conclusions:**

DNA from *Legionella* species was widely present in aerosols collected from outdoor sites near asphalt roads, especially during the rainy season. Our findings suggest that there may be a risk of exposure to *Legionella* species not only in bathrooms but also in the areas surrounding asphalt roads. Therefore, the possibility of contracting legionellosis in daily life should be considered.

**Supplementary Information:**

The online version contains supplementary material available at 10.1186/s12866-021-02275-2.

## Background

*Legionella* is the causative agent of Legionnaires’ disease, a severe form of legionellosis and a potentially fatal pneumonia [1]. At present, 62 *Legionella* species have been identified [2], approximately half of which have been demonstrated to be pathogenic to humans, and the majority is considered as potential human pathogens [3]. *Legionella* species are ubiquitous in natural environments, and they have also been found in artificial environments, such as cooling towers, baths, showers, and decorative fountains [4–7].

Legionellosis can be acquired through the inhalation of aerosolized water contaminated with *Legionella* [8]. Therefore, aquatic facilities are potential sources of sporadic cases and outbreaks of this disease. According to the National Epidemiological Surveillance of Infectious Diseases, public bath facilities are a major source of *Legionella* infections in Japan [9]. However, in many cases, the sources of infection remain unknown. The results of our previous study suggested that puddles on asphalt roads can serve as potential environmental reservoirs of *L. pneumophila* [10]. Several authors have suggested that *Legionella* present in puddles on roads could be spread by moving cars, which would result in the aerosolization of puddle water, especially on rainy days [11–13]. Recently, several studies have attempted to detect *Legionella* species in aerosols released from hot tap water in bathrooms, shower water, and compost [14–16]. These studies revealed that *Legionella* species are present in aerosols derived from these environments. The Coriolis µ, a portable cyclone-based air sampler for liquid medium, has been used to quantify *Legionella* in bioaerosols by quantitative PCR (qPCR) [14]. However, to date, the prevalence of *Legionella* species in aerosols from outdoor sites near asphalt roads has not been analyzed.

Typically, conventional plate culture has been used to detect *Legionella* in clinical and environmental samples. In some cases, amoebic co-culture has been used, because it can resuscitate viable but non-culturable (VBNC) *Legionella* cells [17] and has a higher sensitivity than plate culture method [18]. In addition, 16S rRNA gene amplicon sequencing has been widely used to detect bacterial pathogens in environmental samples, as metagenomic analysis using 16S rRNA genes is a powerful tool for analyzing microbial communities [19].

The main objective of this study was to determine whether *Legionella* species present in aerosols derived from outdoor sites near asphalt roads could be a source of *Legionella* infection. We investigated the prevalence of *Legionella* species in aerosols from outdoor sites near asphalt roads using amoebic co-culture and qPCR methods. Other types of sampling sites were used and compared; public bathrooms were used as high-risk areas for infection since *Legionella* can be frequently found here, and other indoor sites, such as buildings and private homes, were used as low-risk areas. In addition, to better understand the distribution of *Legionella* and the bacterial community in the air samples collected from outdoor and indoor environments, 16S rRNA gene amplicon sequencing was performed.

## Results

### Recovery of ***Legionella*** from spiked samples

In this study, the detection limit of the qPCR method was 10 *Legionella* colony-forming units (CFU) equivalents in 1 m^3^ of air, and the detection limits both of the amoebic co-culture and the plate culture methods were 10^4^ CFU/m^3^, as determined using *Legionella*-spiked samples.

### Prevalence of ***Legionella*** species in air samples

#### Amoebic co-culture

Of the 202 collected air samples, 150 (129 from roads and 21 from bathrooms) were tested by amoebic co-culture (Supplementary Table S1). *Legionella* species were not isolated by amoebic co-culture from any of the 150 air samples. Although 20 *Legionella*-suspected colonies grew on glycine-vancomycin-polymyxin B-cycloheximide (GVPC) agar plates, they also grew on blood agar plates, indicating that they do not belong to the genus *Legionella*. However, 10-fold more *Legionella* DNA was observed in 10 samples (10/150, 6.7%) using qPCR after amoebic co-culture than in identical samples analyzed without co-culture (Supplementary Table S1). PCR amplification of the macrophage infectivity potentiator (*mip*) gene was performed in these samples, and a PCR product was obtained from only one sample collected from a bathroom (*Legionella*-specific 16S rRNA genes, 1.45 log_10_ copies/m^3^) in which the amount of *Legionella* DNA was increased by 5.8 × 10^4^-fold after amoebic co-culture (6.21 log_10_ copies/m^3^). Direct sequencing of the PCR product showed that the *mip* gene sequence in the sample had 92% identity with the *mip* gene of *L. nautarum* using BLAST.

#### qPCR

*Legionella* DNA was detected in 114/151 (75.5%) air samples collected near roads and at all 12 sampling sites (locations A‒L, Supplementary Table S1). *Legionella* DNA was also detected in 15/21 (71.4%) air samples collected from 14/17 bath facilities and 11/30 (36.7%) samples collected from 4/4 other indoor sites. The positivity rates for samples collected near roads and from bathrooms were significantly higher than those for samples collected from indoor sites other than bathrooms (*P* < 0.05; Fisher’s exact test followed by *post hoc* Holm test). The geometric means ± standard deviation (SD) (log_10_ copies/m^3^) of *Legionella*-specific 16S rRNA gene in the *Legionella* DNA-positive samples were 1.80 ± 0.52, 1.82 ± 0.50, and 0.88 ± 0.56 for roads (N = 114), bathrooms (N = 15), and other indoor sites (N = 11), respectively. The values for these three sampling source types were determined to be significantly different by one-way analysis of variance (one-way ANOVA) (*P* < 0.05). Moreover, the Tukey-Kramer method revealed that the amount of *Legionella* DNA was significantly different between the samples from roads and those collected in indoor sites other than bathrooms (*P* < 0.05) (Table 1).

**Table 1:** Prevalence of *Legionella* species in air samples

Sampling site	No. of samples	No. (%) of *Legionella-*positive samples by qPCR^a^	Geometric mean ± SD (log_10_ copies/m^3^) in *Legionella* DNA-positive samples^b^
Road	151	114 (75.5) A	1.80 ± 0.52 C
Bathroom	21	15 (71.4) A	1.82 ± 0.50 CD
Indoor site	30	11 (36.7) B	0.88 ± 0.56 D

^a^Values with different letters are significantly different (*P* < 0.05). Data were analyzed by Fisher’s exact test followed by *post hoc* Holm test.

^b^Values with different letters are significantly different (*P* < 0.05). Data were analyzed by one-way ANOVA followed by Tukey-Kramer post-hoc test for multiple comparisons.

### Geographic characterization of air samples collected from roads

At the 12 sampling sites near asphalt roads (locations A‒L, N = 151), the detection rates of *Legionella* DNA ranged from 60.0 to 93.3% (Supplementary Table S2). The geometric means ± SD (log_10_ copies/m^3^) of *Legionella*-specific 16S rRNA genes in the *Legionella* DNA-positive samples ranged from 1.54 ± 0.66 to 2.03 ± 0.42. The differences in the detection rates according to sampling site and the amount of *Legionella*-specific 16S rRNA genes according to sampling site were not significant by Fisher’s exact test followed by *post hoc* Holm test and one-way ANOVA, respectively (*P* ≥ 0.05).

### Meteorological characterizations of air samples collected from roads

**Fig. 1:**
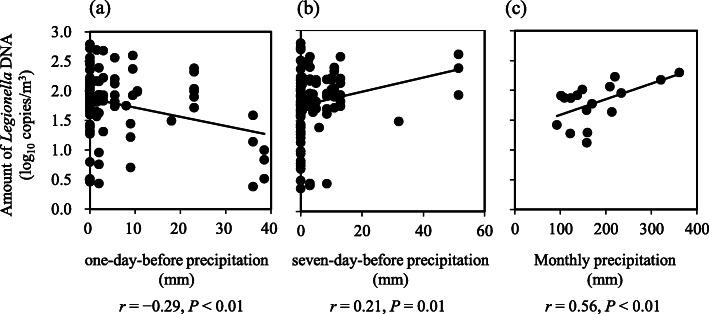
Scatter plots of total precipitation and the amount of *Legionella* DNA. Total precipitation (mm) at each sampling time point and the amount of *Legionella*-specific 16 S rRNA gene (log_10_ copies/m^3^) in the *Legionella* DNA-positive air samples collected near roads in Toyama Prefecture, Japan. (**a**) Daily precipitation one day before sampling (lag day 1). (**b**) Daily precipitation seven days before sampling (lag day 7). (**c**) Monthly precipitation

We assessed the correlation between the climatic conditions (air temperature, relative humidity, total precipitation, and wind speed) and the amount of *Legionella*-specific 16S rRNA genes (log_10_ copies/m^3^) in the *Legionella* DNA-positive samples (Table 2). The amount of *Legionella* DNA was correlated with the monthly total precipitation (N = 17, *r* = 0.56, *P* < 0.01). It was also directly and inversely correlated with the daily total precipitation for seven days (N = 114, *r* = 0.21, *P* = 0.01) and one day (N = 114, *r* = − 0.29, *P* < 0.01) before the sampling day, respectively. The scatter plots of total precipitation and the amount of *Legionella* DNA are shown in Fig. 1. The detection rate and the geometric mean of *Legionella* DNA seven days before the sampling day at a daily total precipitation of > 10 mm (100%, 16/16 samples; 2.07 ± 0.32 log_10_ copies/m^3^, N = 16) were also higher than those at a daily total precipitation of ≤ 10 mm (72.6%, 98/135 samples; 1.75 ± 0.53 log_10_ copies/m^3^, N = 98) (*P* < 0.05; Fisher’s exact test and Student’s *t-test*, respectively). However, the geometric mean of *Legionella* DNA one day before the sampling day at a daily total precipitation of ≤ 10 mm (1.83 ± 0.49 log_10_ copies/m^3^, N = 99) was higher than that at a daily total precipitation of > 10 mm (1.55 ± 0.64 log_10_ copies/m^3^, N = 15; *P* < 0.05, Student’s *t-test*); the detection rates of *Legionella* DNA one day before the sampling day at daily total precipitation of ≤ 10 mm (75.0%, 99/132 samples) and > 10 mm (78.9%, 15/19 samples) were not significantly different (*P* ≥ 0.05, Fisher’s exact test).

**Table 2:** Correlation between the climatic conditions and the amount of *Legionella* DNA^a^

	Air temperature (mean, °C)		Relative humidity (mean, %)		Total precipitation (mm)		Wind speed (mean, m/s)	
	*r*	*P*	*r*	*P*	*r*	*P*	*r*	*P*
Daily value (N = 114):								
on the sampling day (lag day 0)	−0.10	0.22	0.13	0.11	0.17	0.04^c^	−0.10	0.22
one day before the sampling day (lag day 1)	0.01	0.90	−0.19	0.02^c^	−0.29^b^	< 0.01^c^	0.16	0.049^c^
two days before the sampling day (lag day 2)	−0.07	0.39	−0.15	0.06	0.12	0.16	0.08	0.34
three days before the sampling day (lag day 3)	−0.10	0.24	−0.01	0.90	−0.11	0.17	−0.15	0.06
four days before the sampling day (lag day 4)	−0.06	0.48	0.03	0.74	0.02	0.85	−0.19	0.02^c^
five days before the sampling day (lag day 5)	−0.09	0.29	−0.05	0.53	−0.06	0.45	−0.08	0.35
six days before the sampling day (lag day 6)	−0.11	0.17	0.04	0.65	−0.09	0.28	0.07	0.39
seven days before the sampling day (lag day 7)	−0.13	0.12	0.14	0.09	0.21^b^	0.01^c^	−0.07	0.37
Monthly value (N = 17)	−0.08	0.35	0.08	0.34	0.56^b^	< 0.01^c^	−0.04	0.63

^a^Geometric mean (log_10_ copies/m^3^) of *Legionella*-specific 16S rRNA genes in the *Legionella* DNA-positive samples.

^b^An absolute Pearson’s *r* value ≥ 0.20 was considered to be correlated.

^c^*P* < 0.05 was considered significant.

### 16S rRNA gene amplicon sequencing

#### Sequencing analysis

16S rRNA gene amplicon sequencing was performed on randomly selected samples collected from roads (N = 30) and all samples collected from bathrooms (N = 21) and other indoor sites (N = 30). The median number of reads after quality filtering, denoising, merging, and removing chimeric sequences was 62,246 (range, 9,852‒246,625) from roads, 112,471 (range, 16,534‒341,587) from bathrooms, and 111,835 (range, 16,009‒250,862) from other indoor sites. A total of 8,174,054 reads (100,914 reads per sample) were assigned to 18,426 amplicon sequence variants (ASVs).

#### *Legionella*‑assigned reads

Reads from *Legionella* species were detected in 15/81 samples (19%) in total: 9/30 samples (30%) collected near roads (mean proportion of reads, 0.11%), 5/21 samples (24%) collected in bathrooms (mean, 0.04%), and 1/30 samples (3%) collected from indoor sites other than bathrooms (mean, 0.03%). The rate of positivity for samples collected near roads was significantly higher than that for samples collected from indoor sites other than bathrooms (*P* < 0.05; Fisher’s exact test followed by *post hoc* Holm test).

Among the 15 samples in which *Legionella* reads were detected, *Legionella* species was identified in 6 samples (Table 3). At the species level, *L. pneumophila* was identified in 2/30 samples collected near roads (sample no. 22 and 86; the proportion of reads, 0.09 and 0.11% of the total reads number in each positive sample) and in 1/21 samples collected in bathrooms (sample no. 98; 0.15%). *L. birminghamensis* was identified in 1/30 samples collected from roads (sample no. 107, 0.81%). *L. geestiana* was identified in 1/30 samples collected from roads (sample no. 35, 0.02%). *L. nautarum* was identified in 1/30 samples collected from indoor sites other than bathrooms (sample no. 159, 0.79%). The reads in the remaining 9 *Legionella*-positive samples could not be identified at the species level.

**Table 3:** Analysis of air samples containing *Legionella*-assigned reads at the species level

Sample No.	Sampling source type	qPCR (copies/m^3^)	qPCR with amoebic co-culture (copies/m^3^)	No. of reads assigned to:				Total	Number of clean sequences	Detection rate of *Legionella *species (%)
				*L. birminghamensis*	*L. geestiana*	*L. nautarum*	*L. pneumophila*			
22	Road	161.6	0.0	0	0	0	31	31	33,964	0.09
35	Road	245.7	15.8	0	33	0	0	33	185,688	0.02
86	Road	6.3	0.0	0	0	0	122	122	114,647	0.11
98	Bathroom	65.7	0.0	0	0	0	165	165	112,066	0.15
107	Road	365.0	67.7	1743	0	0	0	1743	215,097	0.81
159	Indoor site	72.1	Not tested	0	0	400	0	400	50,889	0.79

#### Characteristics of the bacterial communities

At the genus level, 485, 421, and 368 bacterial genera were detected in samples collected from roads, bathrooms, and other indoor sites, respectively (Supplementary Figure S1). The top three most abundant bacterial genera were *Sphingomonas* (21.1%), *Streptococcus* (14.6%), and *Methylobacterium* (1.6%) in the samples collected near roads; *Sphingomonas* (17.6%), *Pseudomonas* (5.4%), and *Methylococcus* (4.3%) in the samples collected in bathrooms, and *Sphingomonas* (19.2%), *Achromobacter* (5.0%), and *Arthrobacter* (3.8%) in the samples collected from indoor sites other than bathrooms.

**Fig. 2:**
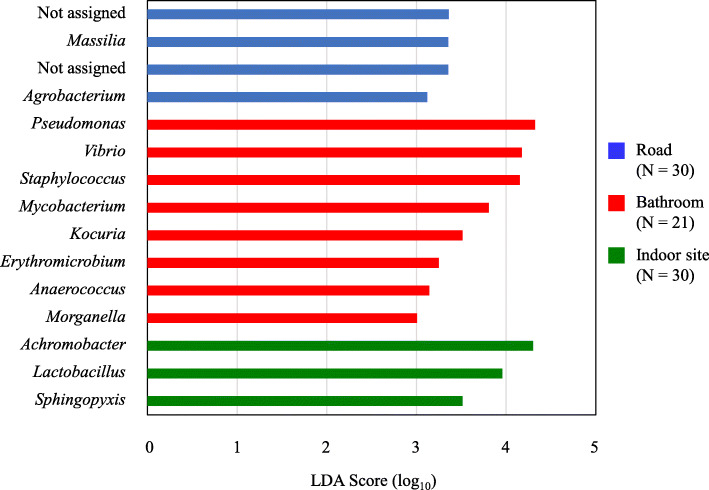
LEfSe analysis. LEfSe identified the most differentially abundant genera among the road (blue bar), bathroom (red bar), and other indoor sites (green bar). A total of 15 bacterial genera were considered significant. The threshold for the logarithmic LDA score is 3.0, with a *P* value less than 0.05 (pairwise Wilcoxon test)

The alpha diversity index based on Faith’s phylogenetic diversity, a qualitative measure of bacterial community richness that incorporates phylogenetic relationships between features, in air samples from roads were much higher value than those from other indoor sites [*Q* < 0.05; Kruskal-Wallis test with Benjamini-Hochberg false discovery rate (FDR) correction] (Supplementary Figure S2). Beta diversity analysis showed that a portion of the air samples collected from roads and indoor sites other than bathrooms were separate from samples collected from bathrooms (Supplementary Figure S3). Linear discriminant analysis (LDA) effect size (LEfSe) analysis to determine the significantly different taxa among the three sampling sources revealed 15 genera with LDA scores of at least 3.0 (Fig. 2; *P* < 0.05, pairwise Wilcoxon test). Specifically, we found that the following four genera were enriched in the samples (LDA score > 4.0): *Pseudomonas*, *Vibrio*, and *Staphylococcus* in samples from bathrooms (shown in red), and *Achromobacter* in samples from other indoor sites (shown in green).

## Discussion

We demonstrated that DNA from *Legionella* species was widely present in aerosols derived from outdoor sites near asphalt roads, especially during the rainy season, regardless of sampling site. *Legionella* DNA was detected in more than 70% of the air samples collected near roads. The positivity rate for samples collected near roads was almost the same as that for samples collected in the bathrooms of public bath facilities, which are a major source of legionellosis in Japan and was significantly higher than that for samples collected from other indoor sites (*P* < 0.05). Similarly, Montagna et al. reported that *Legionella* DNA was detected in 72.7% (8/11) of air samples collected from bathrooms in healthcare facilities using a Coriolis µ air sampler by qPCR [14]. Low-level detection of *Legionella* DNA in air samples collected from other indoor sites (buildings and private homes) may be because airborne bacteria from the outdoor environment enter buildings and houses via ventilation components such as air-conditioning systems [20]. Although the sampling height in this study was not at 150–180 cm above ground level, which is the average height of an adult, owing to the stability of the air sampler, our results showed the existence of aerosols containing *Legionella* species near asphalt roads. Further investigation of the relationship between sampling height and the prevalence of *Legionella* species in aerosols is needed to reveal the risk of inhalation of aerosols containing *Legionella* species.

No *Legionella* species were isolated from any of the collected air samples (roads, bathrooms, and other indoor sites) by amoebic co-culture. The discrepant results for the amoebic co-culture and qPCR methods may be due to the detection limit of each method. Although our control experiment showed that the detection limit of the amoebic co-culture method was comparable to those of the plate culture method, Conza et al. reported that the detection limit of the amoebic co-culture method was 3 logs high sensitive than those of the plate culture method and that viable *Legionella* cells were isolated by amoebic co-culture from aerosols collected at composting facilities [15, 18]. Edagawa et al. reported that a combination of amoebic co-culture and qPCR might be useful to detect viable *Legionella* cells [21]. In this study, 10-fold more *Legionella* DNA was observed in 10 samples after amoebic co-culture, indicating that amoebic co-culture promoted the growth of *Legionella* cells such as VBNC state in the environment. The recovery of *Legionella* from air sample may be due to the state of *Legionella* cells. In addition, it is also possible *Legionella* species may not have been isolated because of the stress encountered during aerosolization and the air sampling process, which may have led to a loss of culturability [22]. In fact, Montagna et al. reported that viable *Legionella* cells in air samples collected using a Coriolis µ air sampler could not be isolated by culture [14]. Improvements in the amoebic co-culture method, such as the strains of amoeba used for co-culture and the incubation periods with the amoeba, may also allow the isolation of *Legionella* from aerosols. Nonetheless, new sampling and/or culture methods need to be established to allow the isolation of *Legionella* species from aerosols.

It seems plausible that precipitation is positively associated with the occurrence of legionellosis [23]. Our study also showed a positive correlation between the monthly total precipitation and the amount of *Legionella* DNA present in aerosols collected near roads. According to previous studies, the number of legionellosis cases in Japan peak in July, which is in the second half of the rainy season [24, 25]. Thus, there is a risk of contracting legionellosis near asphalt roads, especially during the rainy season. The amount of *Legionella* DNA was also directly and inversely correlated with the daily total precipitation of seven days and one day before sampling, respectively. Our results suggest that *Legionella* may have multiplied within amoeba present in the environment after precipitation at the seven-day-before sampling time point, whereas the dry condition during the day before the sampling time point may have favored the release of aerosols of small particle sizes from the ground into the atmosphere because of splashing or the effect of wind. Alternatively, the survival rate of *Legionella* in aerosols may be different due to climatic conditions.

We assessed the distribution of *Legionella* and the bacterial community in air samples using 16S rRNA gene amplicon sequencing. Reads from *Legionella* species were detected in 30% of the air samples collected near roads, which was similar to the proportion of reads detected in bathrooms and was significantly higher than those detected in other indoor sites. *L. pneumophila*, a major causative agent of Legionnaires’ disease [26], was also detected in aerosols collected near roads and in bathrooms. In outdoor environment, other non-*L. pneumophila* species, such as *L. longbeachae*, derived from soil were also important for legionellosis [27], indicating the importance of the detection of non-*L. pneumophila* and species identification. Our results suggest that legionellosis may be acquired by inhaling aerosols from areas near asphalt roads, as well as in bathrooms. The detection rate of *Legionella* reads by 16S rRNA gene amplicon sequencing was lower than that by qPCR (75.5%). This may be due to the PCR amplification bias of the 16S rRNA gene. Conversely, the detection rate and geometric mean of *Legionella* DNA by qPCR for samples in which *Legionella* reads were detected using 16S rRNA gene amplicon sequencing (93.3%, 14/15 samples; 2.04 ± 0.56, log_10_ copies/m^3^) were significantly higher than those in the samples in which *Legionella* reads were not detected by 16S rRNA gene amplicon sequencing (57.6%, 38/66 samples; 1.49 ± 0.56 log_10_ copies/m^3^) (*P* < 0.05; Fisher’s exact test and Student’s *t-test*, respectively). Thus, the qPCR results for *Legionella* DNA were correlated with the 16S rRNA gene amplicon sequencing results.

Our study revealed that the bacterial diversity in outdoor environment was comparable to that in indoor environment which contains aerosol-generating features and higher than that in indoor environment without the features. In addition, the bacterial community composition differed in air samples from outdoor and indoor environments. Then, we analyzed what genera account for the diversity and significance by LEfSe analysis. *Sphingomonas* was the most frequently detected genus in the air samples. This genus has been found in various environments, such as soil, water, clinical specimens, air, and other locations [28–30], indicating the opportunity for these bacteria to be released into the air. *Pseudomonas* and *Achromobacter* were significantly enriched in air samples from bathrooms and other indoor sites, respectively. These genera have frequently been detected in bathwater and moist indoor environments [31, 32]. Thus, our results agreed with those reported in other studies. These findings provide a foundation for understanding the health effects of aerosols in these environments.

In this study, there are several limitations. First, it has not been investigated whether DNA from *Legionella* species detected in air samples is derived from VBNC or dead cells. Although various stress factors may induce *Legionella* cells to enter a VBNC state, these cells can still directly infect human macrophages and amoebae, indicating that VBNC *Legionella* cells can cause disease in humans [33, 34]. Several studies have shown that VBNC *Legionella* in water samples regain culturability in amoebic co-culture [17, 35]. Although *Legionella* is sensitive to environmental stresses such as desiccation and UV irradiation, amoeba cysts, which are resistant amoebal forms that can survive under stress conditions [36], may play a role in the survival and transmission of airborne *Legionella*. Further investigations using techniques such as ethidium monoazide with qPCR, which can selectively quantify viable *Legionella* cells because ethidium monoazide prevents the amplification of DNA from membrane-damaged dead cells [37], are needed to obtain a detailed understanding of the state of *Legionella* in aerosols. Second, regarding the outdoor sites near asphalt roads, information on the sampling sites such as traffic volume at the sampling time and the precise number of people walking on foot on the neighboring was missing. A risk of contracting legionellosis depends not only on the concentration of *Legionella* in aerosols, but also on the exposure to *Legionella* cells. However, the exposure assessment was not investigated in this study. Furthermore, we could investigated only 12 sampling sites. Thus, it remains unclear how the road conditions influenced aerosolization of water. The relevance on the road conditions and *Legionella* prevalence in aerosols seems an interesting topic for future research.

## Conclusions

Here, we demonstrated that DNA from *Legionella* species was widely present in aerosols collected from outdoor sites near asphalt roads, especially during the rainy season, regardless of the sampling site. Our findings suggest that there may be a risk of exposure to *Legionella* species not only in bathrooms but also in the areas surrounding asphalt roads. Therefore, the possibility of contracting legionellosis in daily life should be considered. Further studies are needed to evaluate the risk of legionellosis and develop prevention strategies.

## Methods

### Air samples

We investigated outdoor sites near asphalt roads, indoor sites in bathrooms next to bathtubs in public bath facilities, and other indoor sites in Toyama Prefecture, Japan (Supplementary Table S1). A total of 202 air samples were collected, including 151 samples from 12 outdoor sites (locations A‒L) near asphalt roads, on various days in sunny, cloudy, and rainy weather for almost 1 year from June 2016 to April 2017. The numbers of air samples collected near roads at the 12 sampling sites were as follows: A, N = 15; B, N = 12; C, N = 12; D, N = 11; E, N = 10; F, N = 13; G, N = 11; H, N = 11; I, N = 17; J, N = 13; K, N = 11; and L, N = 15. Traffic volume (vehicles/24 h) was shown in Supplementary Table S2 [38]. Although it was not the data at the time of air sampling, we used them as the latest published data (2015) for reference. These sampling sites were located near the external city roads, and there were several people walking on foot on the neighboring roads during the sampling period.

Twenty-one samples were collected from bathrooms next to bathtubs in 17 public bath facilities from October 2016 to February 2017 (16 were collected while operating aerosol-generating devices in a bathtub, such as jet circulation with or without air induction bubbles). The remaining 30 samples were collected in rooms and corridors from two buildings and two private houses from March 2017 to February 2018 (indoor sites other than bathrooms). At the sites, devices that generate aerosols of water, such as showers and taps, were not operated during the sampling period.

### Sample collection

For each sampling, a portable cyclone-based air sampler (Coriolis µ; Bertin Technologies, France) was placed approximately 70 cm from the ground. The air samples were collected in 15 mL of a solution containing 0.005% Tween 80 at a flow rate of 300 L/min for 10 min. Depending on the climatic conditions, the remaining volume after sampling was 6‒14 mL, and each sample solution was vortexed for 1 min.

### Control experiment

We assessed the detection limits of the culture, amoebic co-culture, and qPCR methods from the air samples as described previously with a slight modification [18]. In this study, air samples were collected in the field with a Coriolis µ under the conditions described above. For our experiments, we used 15 mL of sterilized 0.005% Tween 80 solution spiked with known amounts of *Legionella*. Five 14.7 mL aliquots of sterilized 0.005% Tween 80 were spiked with 0.3 mL of serial dilutions of *L. pneumophila* (ATCC33152) to obtain suspensions containing 3–30,000 CFU in 15 mL of 0.005% Tween 80. Then, a collector cone was filled with the spiked solution and connected to the Coriolis µ. After operating the Coriolis µ at a flow rate of 300 L/min for 10 min in a pre-cleaned biological safety cabinet, the spiked solution was used for culture, amoebic co-culture, and qPCR. Fifteen milliliters of sterile 0.005% Tween 80 solution was used as a negative control. All measurements were performed in duplicate.

### qPCR

For qPCR, DNA was extracted from a 2 mL aliquot of sample solution. The suspension was centrifuged at 20,000 × *g* at room temperature for 5 min and then resuspended in 100 µL of 5% (w/v) Chelex-100 solution ([39]; Bio-Rad Laboratories, CA, USA). The suspension was boiled for 10 min and then centrifuged at 20,000 × *g* for 5 min at room temperature. The supernatant was used as the DNA template, and qPCR was carried out using the CycleavePCR *Legionella* (16S rRNA) Detection Kit (Takara Bio, Shiga, Japan) and a Thermal Cycler Dice Real Time System II (Takara Bio). According to the manufacturer’s instructions, this qPCR assay was positive for 74 *Legionella* strains (48 *Legionella* species) and negative for 14 non-*Legionella* strains (11 non-*Legionella* species: *Shigella sonnei*, *Escherichia coli*, *Vibrio parahaemolyticus*, *Campylobacter jejuni*, *Salmonella enterica*, *Clostridium botulinum*, *Clostridium perfringens*, *Staphylococcus aureus*, *Yersinia enterocolitica*, *Listeria monocytogenes*, and *Bacillus cereus*), and 1 CFU of *L. pneumophila* corresponded to 23 copies of 16S rRNA genes, which was calculated from DNA standard curve.

### Isolation of ***Legionella*** species

Amoebic co-culture was performed as described previously [40]. *Acanthamoeba* species isolated from cooling tower water were incubated in proteose peptone-yeast extract-glucose-cysteine medium at 30°C for 5‒7 days. Cells were washed and resuspended in phosphate-buffered saline, and then 0.5 mL of the suspension (approximately 1.0 × 10^5^ cells) was added to the remaining amount of the sampling solution (4‒12 mL) after qPCR sampling (2 mL). To prevent evaporation, the samples were incubated at 35°C in 50 mL screw-cap tubes. After 4 weeks, the sample was mixed with equal volumes of 0.2 mol/L KCl-HCl buffer (pH 2.2) for 15 min at room temperature, and 0.2 mL was spread on a GVPC agar plate (Nissui Pharmaceutical Co., Tokyo, Japan). The agar plates were incubated at 35 °C for 7 days in a humidified chamber. Candidate colonies that were smooth gray with characteristic outward structures of *Legionella*, i.e., cut-glass-like or mosaic-like in appearance were viewed under a stereomicroscope with oblique illumination [41] and were subcultured on a buffered charcoal-yeast extract (BCYE) agar plate with L-cysteine (bioMérieux, Lyon, France) and a blood agar plate (Eiken Chemical, Tokyo, Japan). Colonies growing only on the BCYE agar plate and not on the blood agar plate were presumed to belong to the genus *Legionella*.

### Sequencing of the ***mip*** gene

For samples in which the amount of *Legionella* DNA increased after amoebic co-culture, the species of *Legionella* was determined. DNA was extracted from a 2 mL sample of the amoebic co-culture using Chelex-100 solution as described above. The *mip* gene was directly amplified and sequenced as previously described [42].

### Next-generation sequencing for 16S rRNA gene amplicon

DNA samples extracted with Chelex-100 solution were used for next-generation sequencing. For air samples randomly selected from roads (N = 30) and collected from bathrooms (N = 21) and other indoor sites (N = 30), the bacterial 16 S V3–4 region was amplified using Tks Gflex DNA Polymerase (Takara Bio) with the primers 341F (5′-TCGTCGGCAGCGTCAGATGTGTATAAGAGACAG-3′) and 806R (5′-GTCTCGTGGGCTCGGAGATGTGTATAAGAGACAG-3′). A 16S rRNA gene amplicon library was prepared according to the 16S Metagenomic Sequencing Library Preparation protocol for Illumina MiSeq (Illumina, San Diego, CA, USA) and then sequenced using an Illumina MiSeq instrument with the MiSeq Reagent Kit v3 (600 cycles).

### Bioinformatic processing

Microbiome bioinformatics was performed using QIIME2 version 2019.7 [43]. Imported demultiplexed sequence data were denoised using DADA2 [44] (via q2-dada2). All ASVs were aligned with mafft [45] (via q2-alignment), which was used to construct a phylogeny with fasttree2 [46] (via q2-phylogeny). Taxonomy, from kingdom to species, was assigned to ASVs using the q2-feature-classifier [47] based on the classify-sklearn naïve Bayes taxonomy classifier against the GreenGenes 13_8 99 % OTU database [48]. However, the short fragments generated by 16S rRNA amplicon sequencing (< 500 bp in this study) limit their use for 16S rRNA gene-based bacterial identification at the species level due to the sequence similarity of the variable regions [49]. Alpha (Faith’s phylogenetic diversity [50]) and beta diversity metrics (weighted UniFrac [51]) were performed to assess the diversity of the microbial communities in the air samples. These metrics were estimated using q2-diversity after samples were rarefied (subsampled without replacement) to 9,852 sequences per sample. The coordinate points of beta diversity were shown in Supplementary Table S3. Different taxa with significant differences between sampling source types were assessed using the LEfSe program with the following parameters (alpha value for pairwise Wilcoxon test set at 0.05; the logarithmic LDA score threshold was set at 3.0) using the Galaxy Hutlab online platform [52] (https://huttenhower.sph.harvard.edu/galaxy/).

### Statistical analysis

To investigate the relationships between the rate of *Legionella* DNA detection and sampling source type and between the detection rate and sampling site (locations A‒L), Fisher’s exact test followed by *post hoc* Holm test were performed. Fisher’s exact test was also used to compare the rates of *Legionella* DNA detection in samples according to total precipitation (high and low). We also investigated the relationships between the amount of *Legionella* DNA and sampling source type and between the amount of *Legionella* DNA and sampling site (locations A‒L) using one-way ANOVA followed by Tukey–Kramer multiple comparisons. Student’s *t-test* was performed to compare the amount of *Legionella* DNA between samples with high and low total precipitation. These tests were performed using the R statistical software package (version 3.0.0). Pearson’s correlation coefficient (*r*) between climatic conditions (air temperature, relative humidity, total precipitation, and wind speed) and the amount of *Legionella* DNA was calculated using Excel (Microsoft, Redmond, WA, USA). An absolute Pearson’s *r* value ≥ 0.20 was considered indicative of a correlation, and a *P* value less than 0.05 was considered significant. The Kruskal-Wallis test with Benjamini-Hochberg FDR correction (*Q* value [53]) was performed to compare alpha diversity indices using QIIME2. The *Q* value cutoff was set to 0.05.

### Meteorological data collection

During the study period (2016‒2018), meteorological data were obtained from two main weather stations in Fushiki and Toyama, Toyama Prefecture, Japan [54]. The meteorological data used in this study were monthly or daily values of air temperature (mean, °C), relative humidity (mean, %), total precipitation (mm), and wind speed (mean, m/s). To consider the period for *Legionella* growth in the environment, the amount of *Legionella* DNA (log_10_ copies/m^3^) and the daily meteorological values for lag day- 0 to 7 were compared. To analyze seasonal trends, the amount of *Legionella* DNA (log_10_ copies/m^3^) and monthly values were compared (Supplementary Table S4).

## Supplementary Information

**Additional file 1.**

**Additional file 2.**

## Data Availability

The datasets supporting the conclusions of this article are deposited in the DNA Data Bank of Japan (http://www.ddbj.nig.ac.jp/) under accession numbers LC472487 (http://getentry.ddbj.nig.ac.jp/getentry/na/LC472487/?format=flatfile&filetype=html&trace=true&show_suppressed=false&limit=10) for the *mip* gene and DRA008310 (https://ddbj.nig.ac.jp/DRASearch/submission?acc=DRA008310) and DRA009422 (https://ddbj.nig.ac.jp/DRASearch/submission?acc=DRA009422) for the 16 S rRNA gene amplicons. The data deposited is publicly available.

## Abbreviations

qPCR: Quantitative PCR
GVPC: Glycine-vancomycin-polymyxin B-cycloheximide
CFU: Colony-forming units
SD: Standard deviation
ANOVA: Analysis of variance
mip: Macrophage infectivity potentiator
VBNC: Viable but non-culturable
ASV: Amplicon sequence variants
LDA: Linear discriminant analysis
LEfSe: Linear discriminant analysis effect size
BCYE: Buffered charcoal-yeast extract
FDR: False discovery rate
