# Supplementary material for: Detection of Legionella species, the influence of precipitation on the amount of Legionella DNA, and bacterial microbiome in aerosols from outdoor sites near asphalt roads in Toyama Prefecture, Japan
Source: BMC Microbiol. 2021 Jul 17;21:215. doi: 10.1186/s12866-021-02275-2 (PMC8285874; doi:10.1186/s12866-021-02275-2)
Supplement: Supplementary file 2 — Additional file 2. [file 12866_2021_2275_MOESM2_ESM.pptx]

## Slide 1
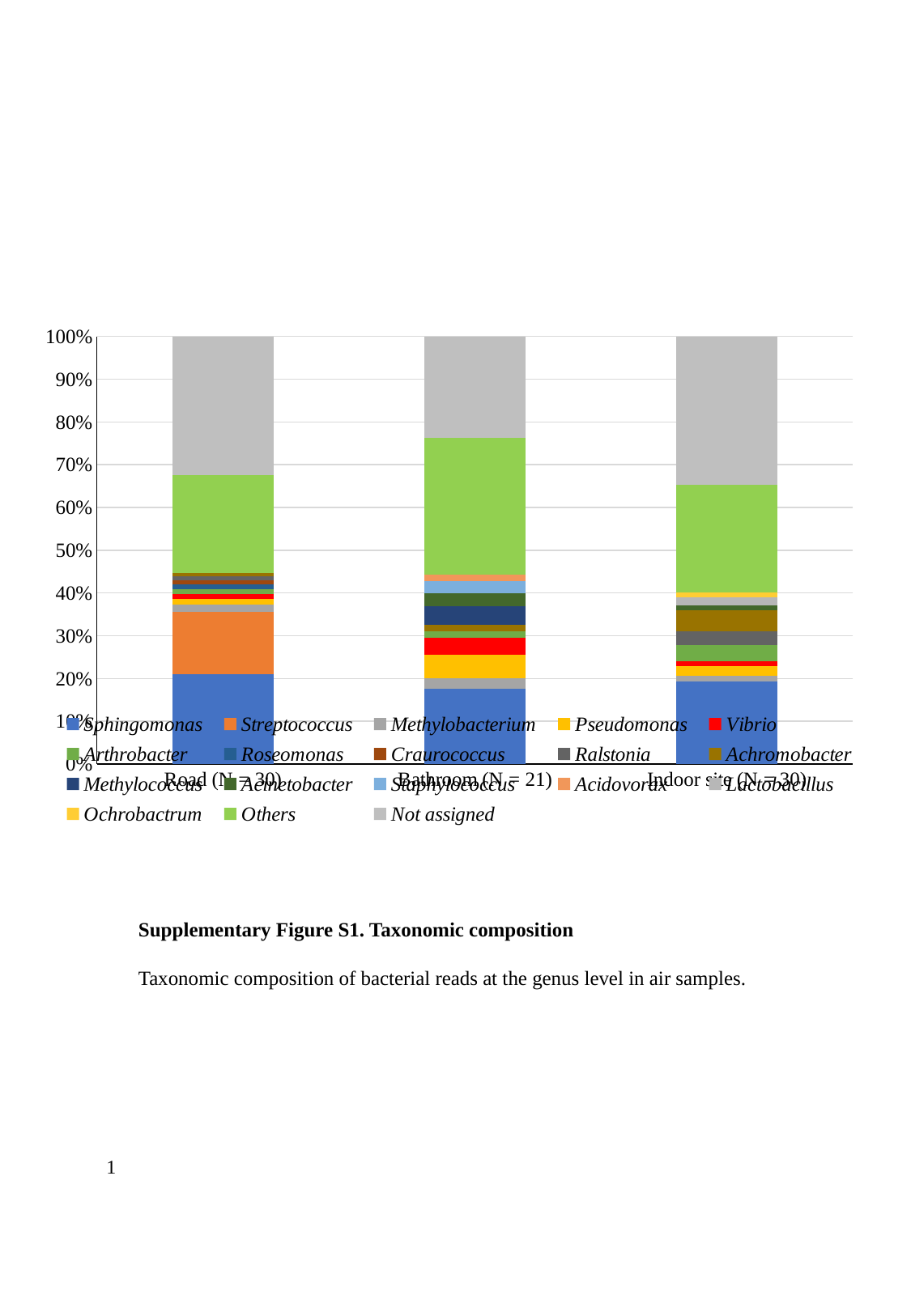

### Chart
| Category | Sphingomonas | Streptococcus | Methylobacterium | Pseudomonas | Vibrio | Arthrobacter | Roseomonas | Craurococcus | Ralstonia | Achromobacter | Methylococcus | Acinetobacter | Staphylococcus | Acidovorax | Lactobacillus | Ochrobactrum | Others | Not assigned |
|---|---|---|---|---|---|---|---|---|---|---|---|---|---|---|---|---|---|---|
| Road (N = 30) | 21.0501031033333 | 14.60445911 | 1.56865388 | 1.3720205499999998 | 1.2333374666666665 | 1.0806349566666666 | 1.0142657199999996 | 1.0098573633333334 | 0.9684033866666666 | 0.8329542400000001 | 0.0 | 0.0 | 0.0 | 0.0 | 0.0 | 0.0 | 22.90250768 | 32.36280308 |
| Bathroom (N = 21) | 17.583036214285713 | 0.0 | 2.5676659285714294 | 5.446565052380955 | 3.867302890476191 | 1.6169958952380954 | 0.0 | 0.0 | 0.0 | 1.539334142857143 | 4.275058466666666 | 3.018473919047619 | 2.787724238095238 | 1.5567082238095236 | 0.0 | 0.0 | 31.930984080952364 | 23.81014972857143 |
| Indoor site (N = 30) | 19.24941342333333 | 0.0 | 1.4287131733333336 | 2.2867768099999997 | 1.0790293399999995 | 3.8162108533333328 | 0.0 | 0.0 | 3.0958841466666667 | 5.02916668 | 0.0 | 1.2 | 0.0 | 0.0 | 1.7554757166666668 | 1.2685842633333335 | 25.00507185999997 | 34.78383027000001 |Supplementary Figure S1. Taxonomic composition
Taxonomic composition of bacterial reads at the genus level in air samples.
1

## Slide 2
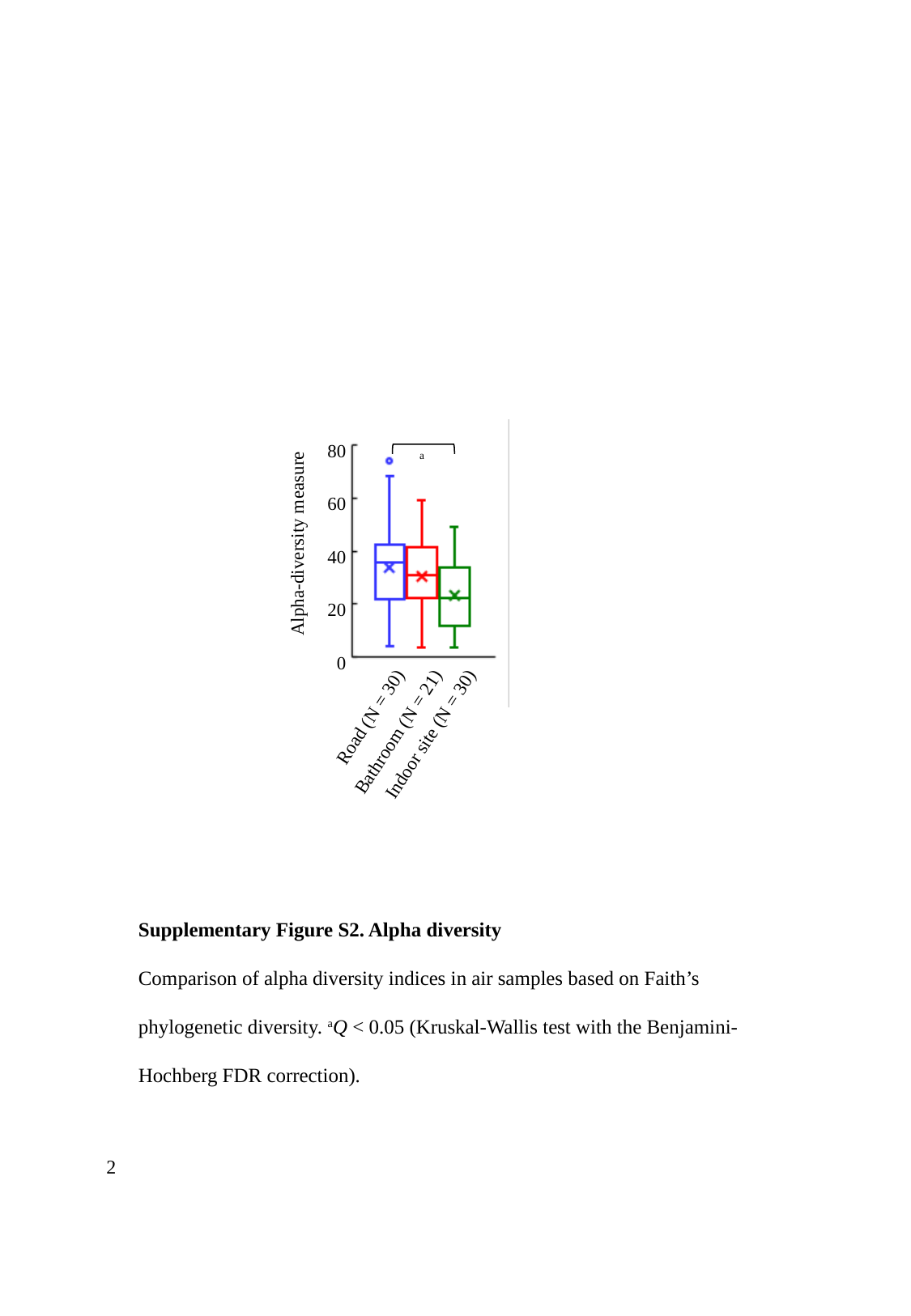

80
a
60
Alpha-diversity measure
40
20
0
Road (N = 30)
Bathroom (N = 21)
Indoor site (N = 30)
Supplementary Figure S2. Alpha diversity
Comparison of alpha diversity indices in air samples based on Faith’s phylogenetic diversity. aQ < 0.05 (Kruskal-Wallis test with the Benjamini-Hochberg FDR correction).
2

## Slide 3
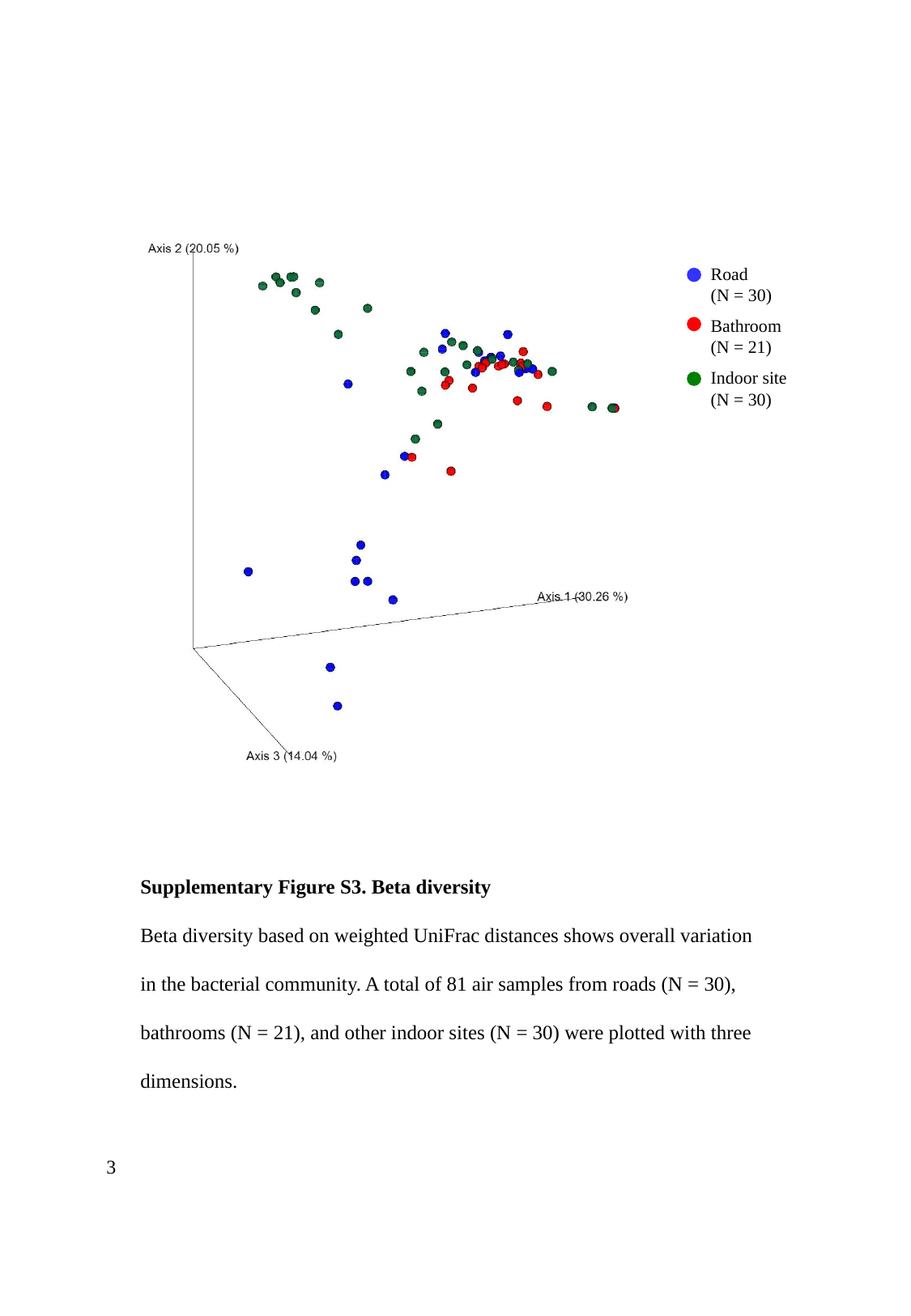

Road
(N = 30)
Bathroom
(N = 21)
Indoor site
(N = 30)
Supplementary Figure S3. Beta diversity
Beta diversity based on weighted UniFrac distances shows overall variation in the bacterial community. A total of 81 air samples from roads (N = 30), bathrooms (N = 21), and other indoor sites (N = 30) were plotted with three dimensions.
3
